# User involvement in digital mental health: approaches, potential and the need for guidelines

**DOI:** 10.3389/fdgth.2024.1440660

**Published:** 2024-08-22

**Authors:** Sylvie Bernaerts, Tom Van Daele, Christian Korthé Carlsen, Søren Lange Nielsen, Jolanda Schaap, Yvette Roke

**Affiliations:** ^1^ Psychology and Technology, Centre of Expertise Care and Well-Being, Thomas More University of Applied Sciences, Antwerp, Belgium; ^2^ Centre for Technological Innovation, Mental Health and Education, Queen's University Belfast, Belfast, United Kingdom; ^3^ Centre for Digital Psychiatry, Region of Southern Denmark, Odense, Denmark; ^4^ Expertise Center for Autism Spectrum Disorder, GGZ Centraal, Almere, Netherlands

**Keywords:** digital mental health, user participation, end-user involvement, guidelines, adaptation

## Introduction

Over the past decades, the importance of mental health is increasingly being acknowledged, with more people reaching out for help. However, mental healthcare struggles to help all in need. Those finding their way to formal services face long waiting lists, while for others, the associated stigma is still too large to reach out. Both cases result in unmet needs, which remain a pressing issue. One attempt to overcome these challenges is to rely on digital mental health, the use of technology for mental health interventions, ranging from promotion, prevention, and treatment to maintenance. Technologies can for example include computers and smartphones, extended reality, wearables, social media, chatbots (1) and may or may not make use of artificial intelligence. A wealth of evidence already supports the efficacy and effectiveness of online interventions for common mental health conditions, such as depression and anxiety in older adults (2), and in adolescents and young people (3) as well as – to a lesser extent - their cost-effectiveness (4). Despite this potential, successful implementation of these interventions and other forms of digital mental health has proven to be challenging, particularly concerning adaptation, uptake and adherence (5). As this is a multifaceted challenge a single solution is non-existent. Addressing this challenge requires taking into account many aspects and perspectives, with implementation sciences gaining increased attention as a result. In this opinion paper, we highlight user involvement as one important aspect in the development, implementation and international adaptation of digital mental health interventions which, to date, still often seems to be overlooked. In the following paragraphs we will define the concept, highlight the potential of involving users to facilitate uptake of digital mental health, and argue for the need for clear guidelines on how to do so, not only for initial development, but also for subsequent international adaptation.

## Approaches

In principle, user involvement means that different stakeholders or users are included in one or more steps of the design process. In a context of digital mental health interventions (DMHIs), users might entail patients or clients and their friends and family, clinicians, mental health organizations, and developers. There are, however, multiple approaches to involving these users, of which we highlight three methods: user- centred design, participatory design (or co-design), and user innovation. Within these approaches, the extent to which users have control over design decisions varies: low in user-centred design, higher in participatory design (or co-design), and the highest in user innovation (6). According to Mao et al. (7), user-centred design is a multidisciplinary design approach that actively involves users to improve the understanding of both user requirements and task requirements, as well as the iteration of design and evaluation. In co-design, (potential) users are invited to cooperate with designers, researchers and developers in an innovation process starting from idea generation to decision making (8). Although both approaches are very similar, the difference lies in the starting point and in the extent of the user involvement. In user-centred design, users act as consultants for the designers (after a design idea has already been formulated) and provide feedback throughout the design process. In co-design, users are considered as partners throughout the process from need exploration and idea generation onwards, which ensures that user's' needs and preferences are met and that technologies are acceptable and helpful (9, 10). Finally, in user innovation, the design and development of new products or services is started by end-users, either individual end- users or intermediate users (e.g., organisations) (11). According to a systematic review by Moore et al. (12) user-centred design has been the most reported approach to involve end- users in digital health innovations. However, co-design can be put forward as the more sensible approach to involve vulnerable populations, such as children (9) and older adults (13), but also individuals with mental health conditions (14). Specific methods range from brief user consultation through a review process all the way up to true collaboration. There is not one clear method or process of user involvement, yet common methods include focus groups, surveys, interviews, prototype/storyboards, think-aloud exercises and literature search (12). In particular, Sanz et al. (15) have shown that studies using co-design methods, mostly rely on interviews and workshops, followed by meetings and surveys. In sum, although there is not a single method to involve users, the most promising and sensible approach seems to be co- design: involving users as partners rather than mere consultants from the onset of the design process (9, 13, 16).

## Potential

Throughout the years, multiple reviews highlighting the relevance of user involvement during the development of DMHIs have been conducted and the results have shown both similarities and differences.

Torous et al. (14) theorized that low DMHI engagement could be due to poor usability, lack of user-centric design and/or a lack of trust (among other reasons), suggesting co-design with users as a potential solution. Indeed, involving different users in the development and implementation of DMHIs can limit known barriers to uptake and engagement. For example, Liverpool et al. (17) have shown that child and youth engagement with DMHIs is influenced by intervention- specific factors, such as suitability, usability and acceptability on the one hand, and person- specific factors, such as motivation, opportunity and capability.

Similarly, a review by Borghouts et al. (18) has also identified barriers and facilitators to user engagement with DMHIs, user-related (e.g., beliefs, experience and skills), as well as intervention-related (e.g., content, perceived fit and usefulness) barriers and facilitators to user engagement with DMHIs. All of these can be enhanced or tackled, respectively, by involving users in a co-design process.

Orlowski et al. (19) found that user involvement (named consumer consultation in their study) helped to shape specific DMHIs for youth, but they also stated that the effects of user involvement in intervention design are unclear due to limited evidence on specific outcomes and insufficient implementation after piloting in research. In addition, Fischer et al. (13) revealed that involving older adults in technology design (not limited to DMH) leads to better learning of the user's needs, designs adjusted to these needs and better quality of the design, but also showed that effects on acceptance and uptake are unclear. In line with these reviews, the findings of Bevan Jones et al. (9) corroborate the notion that there is little evidence on the impact of user involvement on uptake, adherence and intervention effectiveness.

In contrast, a more recent review has shown positive effects of user involvement, in particular, to enhance cultural sensitivity, enrich ideas, increase acceptance of the DMHIs, better engagement and a sense of community (16). Taken together, while research is unclear on specific outcomes concerning uptake and efficacy, findings do support the importance of involving users in a co-design process to tackle barriers and enhance facilitators to uptake and engagement.

### International adaptation of digital mental health interventions

One promising application for user involvement is in the context of international adaptation. Considering the vast number of available DMHIs, in particular mental health apps (14), it is more sensible to use resources to adapt existing, evidence-based DMHIs for use in other countries, rather than to reinvent the wheel. In this respect, adapting interventions to the proposed target population has been a longstanding recommendation (20). Developing or designing technologies to be used beyond a country's borders, however, requires particular considerations further than mere translation of the particular intervention's content. Involving users in the adaptation process can, for example, help to inform about potential user characteristics that may be associated with lower adherence and/or higher drop-out rates (21).

One's approach should therefore take into consideration the target population's cultural, clinical and regulatory aspects, to name only a few. For example, the US has been dominating the app market for smartphone-based mental health apps (22). This means that most apps, evidence-based or not, are primarily in English and developed within the US context, adhering to local regulations and referencing local services. Given that user engagement with a DMHI is enhanced by perceived fit – how well users feel the intervention has culturally appropriate content and understandable language (18) – potential users from Europe, Africa, or Asia might be less interested due to language barriers and lack of cultural sensitivity. To the best of our knowledge, however, evidence on best practices for international adaptation of digital mental health interventions is limited, more so since clear methods for developing and implementing apps in broader international contexts are scarce. In one example, Storm et al. (23) conducted usability tests of an American prototype app called PeerTECH, a peer support app for individuals with a serious mental health condition, with Norwegian users, including clients, clinicians and peer support workers. By doing so, researchers learned that app's adaptation to the Norwegian context would be viable and useful. However, no information on concrete development steps was provided. In another example, Bartlett et al. (24) assessed how well an Australian company involved Arabic-speaking refugees, refugee advocates and healthcare workers during a design thinking process. Their goal was to develop a web-based application to deliver local, evidence-based and culturally relevant health information to its non-English speaking users. Based on their results, relevant recommendations were suggested concerning key communication principles to take into account. Nevertheless, a structured approach for practitionersor researchers to involveusers from different cultures was not discussed. We therefore argue that more research assessing international user involvement is necessary to inspire concrete guidelines for development and international adaptation of digital mental health interventions.

## Need for guidelines

Although researchers have provided frameworks, recommendations and specific methods to involve users, this information seems to be insufficiently specific, nor easily retrievable for entrepreneurs and mental health organizations to use. There is, therefore, a clear need for practice- oriented guidelines aimed at stakeholders on different levels, such as policy makers, entrepreneurs and mental health organizations, on how to involve these different users and mental health professionals in the development, implementation and adaptation of mental health technology.

The formulation of these guidelines entails the consideration of multiple critical factors, of which we will highlight four. As a first point, guidelines require more consistency in terminology. Literature on user involvement mentions the concepts of co-design, co-production, co-creation, participatory design, user involvement, etc, seemingly interchangeably. Although these concepts each have their own definitions, and their operationalisation sometimes also differs, the underlying notion is the same, namely (the importance of) involving different stakeholders, specifically users. A second point involves the need for a comprehensive framework. Similar to the terminology, there are multiple frameworks or theories describing how to involve users, for example, the British Design's Double Diamond model (25), or the Generative Co-Design Framework for Healthcare Innovation (26). No study to date, however, has appeared to have described the use of the Double Diamond model for involving users to design a DMHI. In addition, citation analysis shows that the latter has mainly been referenced for its description of co-design principles rather than for following the steps of the framework itself. In one example, the StigmaBeat project has adopted the framework to involve marginalized youngsters to develop short films for reducing mental health stigma (27). In another example, parents of children with cancer were involved in the co-design process of a paediatric cancer pain management app (28). However, in both cases, no information is provided on resulting adoption or user engagement. A third point of attention constitutes the gaps in evidence concerning development of digital mental health interventions. A recent review by Brotherdale et al. (16) on co-production for digital mental health interventions revealed that there is considerable variability concerning which users to involve, the stage and role of their involvement, which methods are used, which frameworks are implemented and how to deal with power dynamics between designers or producers and users, making it difficult to provide evidence-based guidelines. Notwithstanding these gaps, Brotherdale et al. (16) have also identified several commonalities among studies. Successful involvement of users is often hindered by resource constraints, recruitment challenges, conflicting views within the stakeholders and power imbalances between users and designers. It is, therefore, important to suggest potential (evidence-based) solutions and clearly defined steps on how to tackle these barriers. As a fourth and final point, there are, to the best of our knowledge, no evidence-based recommendations for international adaptation of available digital mental health interventions. It is, however, essential to involve local stakeholders as cultural and regulatory variations between nations are plausible. In light of these evolutions, one initiative that aims to contribute to the aforementioned challenges is the “Successful User Participation Examples and Recommendations”-project (SUPER). Funded by Interreg North Sea Region, it aims to develop guidelines for entrepreneurs and mental health organizations on how to involve different stakeholders, in particular users such as patients and mental health professionals, in the (transnational) development, implementation and adaptation of mental health technology.

## Conclusion

Successful implementation of digital mental health interventions has proven challenging, and in this opinion paper we wanted to argue that user involvement has the potential to provide at least part of the solution. Although evidence on the impact of user involvement on intervention effectiveness is lacking, its added value for increasing cultural sensitivity, enriching ideas, and increasing acceptance of the digital mental health interventions, and improve engagement is clear (16). Nevertheless, translation to practice is hampered by the fact that clear user involvement steps are rarely properly documented and reported in research. Moreover, concrete evidence-based (or even evidence-inspired) guidelines and steps are lacking, making it difficult for practitioners, developers, and healthcare organizations to adequately involve relevant stakeholders in the design and development process, as well in the increasingly common international adaptation of digital mental health applications. Initiatives, such as the SUPER project, are currently underway to help offer a concrete framework and guidelines. Nevertheless, this will still require uptake in research, as well as practice, to lead to improved user involvement and, ideally, also better digital mental health.
